# Influence of cross-sectional geometry on the wave attenuation performance of floating breakwaters: an experimental study

**DOI:** 10.1038/s41598-026-65135-x

**Published:** 2026-08-08

**Authors:** Aya Ashraf, Riham Ezzeldin, Mahmoud E. Saad

**Affiliations:** 1 Irrigation and Hydraulics Engineering Department, Faculty of Engineering, Mansoura University, Mansoura, Egypt; 2 Faculty of Engineering, Mansoura National University, Mansoura, Egypt; 3 Civil Engineering Department, Higher Institute for Engineering and Technology at Manzala, Manzala, Egypt

**Keywords:** Floating breakwaters, Wave attenuation, Coastal protection, Cross-sectional geometry, Triangular profile, Hydrodynamic performance, Trapezoidal profile, Engineering, Physics

## Abstract

Floating breakwaters (FBWs) offer a sustainable alternative for coastal protection against wave-induced erosion. However, the influence of cross-sectional geometry on attenuation performance remains insufficiently quantified. Therefore, this study experimentally investigates the hydrodynamic performance of modular FBWs, focusing on the wave transmission across three distinct profiles: rectangular, triangular, and trapezoidal. These geometries were selected to systematically evaluate the effect of slope angle and wave-surface interaction on wave attenuation. The tested rectangular FBW exhibited a baseline wave attenuation efficiency of 47%, which was systematically improved through geometric modification. Among the three tested inclination angles (α = 30°, 45°, and 60°), the triangular FBW achieved its optimal performance at 30°, reaching an average efficiency of 66%, representing a 19% improvement over the rectangular FBW while requiring 56% less material. The trapezoidal profile demonstrated the highest performance, reaching an average efficiency of 83%. Furthermore, geometric orientation dictates the hydrodynamic mechanism; an upright side-slope configuration outperformed the inverted orientation. In addition, positioning the sloped face on the leeward side enhanced wave attenuation compared to seaward placement. Consequently, a trapezoidal profile with a leeward-oriented slope is recommended for practical implementation because it offers the best balance between hydrodynamic performance, inherent hydrostatic stability, and constructability.

## Introduction

Coastal zones are increasingly vulnerable to wave action, storm surges, and erosion, posing serious risks to infrastructure, ecosystems, and urban communities. Consequently, the development of efficient and sustainable shore protection structures has become a critical priority in modern coastal engineering. For instance, the Egyptian Mediterranean coast has experienced severe shoreline retreat in recent decades, prompting the implementation of various protective measures including jetties, groins, and breakwaters^1^. Within this context, breakwater is an essential structure for protecting the inner water and coastal shoreline and has been used extensively in recent decades^2^. Breakwaters serve as a primary defense mechanism to mitigate wave impacts and maintain shoreline stability by dissipating incident wave energy before it reaches vulnerable coastal zones^3^.

Upon encountering a breakwater, the propagating incident wave energy is partitioned into three components: reflection, transmission, and dissipation. Enhanced wave reflection and energy dissipation systematically lead to a reduction in the transmitted wave height. Consequently, a lower transmission coefficient directly indicates superior wave attenuation performance of the structure^4^.Beyond erosion control, these structures are essential for creating sheltered waters within port basins to facilitate safe ship maneuvering, mooring, and navigation, while simultaneously managing localized sediment transport to prevent the siltation of navigation channels^5^. The degree of protection afforded by breakwaters to the coast is usually measured by the transmission co-efficient, K_t_ defined as the ratio of transmitted to incident wave height^6^.Moreover, the wave attenuation efficiency of breakwaters is typically assessed using the transmission coefficient (K_t_)^7^.

The hydraulic performance of any breakwater is intrinsically related to its cross-sectional configuration and structural classification, which dictate its wave dissipation mechanisms^8^. Traditional coastal defense relying on fixed structures, such as rubble mound and vertical caisson breakwaters has historically demonstrated high wave attenuation performance in nearshore, shallow-water environments^9^. Rubble-mound breakwaters achieve energy dissipation through wave breaking and induced turbulence across permeable slopes^10^, whereas vertical caissons rely primarily on reflecting incident wave energy back into the open sea^11^. However, the deployment of these conventional fixed barriers in deeper water becomes prohibitively expensive and can cause severe environmental impacts, including the degradation of marine ecosystems and the alteration of natural littoral drift patterns^12^.

To circumvent these limitations, floating breakwaters (FBWs) have emerged as a highly adaptable alternative, particularly suited for sites characterized by deep water, poor seabed bearing capacity, or strict environmental regulations. FBWs offer distinct advantages in terms of lower construction costs, ease of installation, and mobility. However, their wave attenuation efficiency is lower than that of fixed breakwaters under severe wave climates^13^. This performance deficit implies that the hydrodynamic success of an FBW heavily depends on the optimum selection of its design parameters, most notably its cross-sectional geometry, draft, freeboard, and mooring stiffness.

Extensive research has been dedicated to study FBW geometric configuration to enhance their hydrodynamic efficiency. Early studies by Sannasiraj et al.,^14^ demonstrated that structural configuration directly governs wave-structure interaction. Also, both the width and the draught of the FB have an effect on its performance. However, the draught seems to be more important than the width^15^. On the other hand, increasing relative width leads to a pronounced reduction in K_t_^16^. For instance, Ji et al.,^17^ demonstrated that when the ratio of breakwater width to wavelength exceeds 0.321, the transmission coefficient remains below 0.5, indicating markedly improved attenuation performance.

Dong^18^, investigated the hydrodynamic performance of three types of floating breakwaters and focusing on their transmission coefficients under regular wave conditions both with and without currents. The results illustrated that; the broad-net design may be adopted for aquaculture engineering in deep-water regions.Investigations illustrated that, geometric modification such as adjusting side slopes or using specialized appendages, can reduce wave transmission by up to 25% under moderate wave climate^19^. More recent innovations include the integration of wing plates to increase wave attenuation performance^20^, whileShen et al.^21^ provided comprehensive insights into performance trends across a wide range of floating breakwater configurations. Paotonan et al.,^22^ illustrated that the key performance indicator of floating breakwaters is wave transmission (K_t_), which is influenced by both structural and wave parameters. Also, Ridlwan et al.,^23^ studied the influence of the frontal face configuration of a floating breakwater on wave transmission, concluding that the square notch structure exhibited the lowest transmission coefficient of 0.624. Furthermore, altering the plan shape of pile-restrained floating breakwaters can significantly enhance wave attenuation, in some cases by up to 60%^24^. Significant improvements have also been achieved through geometry tuning and the integration of energy-loss features such as arc-shaped plates^25^. Incorporating trapezoidal, triangular, and L-shaped appendages can effectively minimize wave transmission while improving overall structural stability^26^. Similarly, numerical and experimental studies on hybrid or multibody configurations have also reported substantial reductions in transmission coefficients, further demonstrating the potential of geometry-based innovations to enhance hydrodynamic performance^27–29^.

Cumulatively, the literature indicates that inclined or non-rectangular modifications dissipate wave energy more effectively than conventional flat-bottomed rectangular designs by redirecting wave momentum without requiring an increase in the structural draft^30^. However, the systematic effect of slope orientation, placement, and inclination angle on wave transmission remains insufficiently quantified, representing the core knowledge gap addressed in this study.

## Research objectives

Building upon previous literature, the primary objective of the present study is to systematically determine the hydrodynamic efficacy of altering the breakwater’s frontal geometry and to identify the optimum design configuration. Specifically, this investigation evaluates whether a sloped surface yields superior wave attenuation when oriented upward or downward, and whether its spatial deployment is more effective at the seaward front or the leeward rear. Furthermore, this work establishes a direct performance benchmark to quantify whether a distinct, hydrodynamic advantage exists when transitioning from a conventional rectangular breakwater to a modified trapezoidal configuration across various inclination angles (α = 30°, 45°, and 60°). By testing these coupled parameters, this study aims to provide coastal engineers with a definitive optimization framework for modular floating breakwaters under diverse wave conditions. Since the transmission coefficient serves as the key metric for assessing the wave attenuation performance of floating breakwaters^31^, this paper focuses on the impact of geometric cross-sections and wave parameters on the transmission coefficient (k_t_).

## Experimental approach

A series of experimental tests was conducted to evaluate the hydrodynamic performance of the rectangular floating breakwater and the impact of shape modification under various wave conditions and structural configurations. The following section describes the experimental approach and the models used in this study.

### The wave flume

**Fig. 1:**
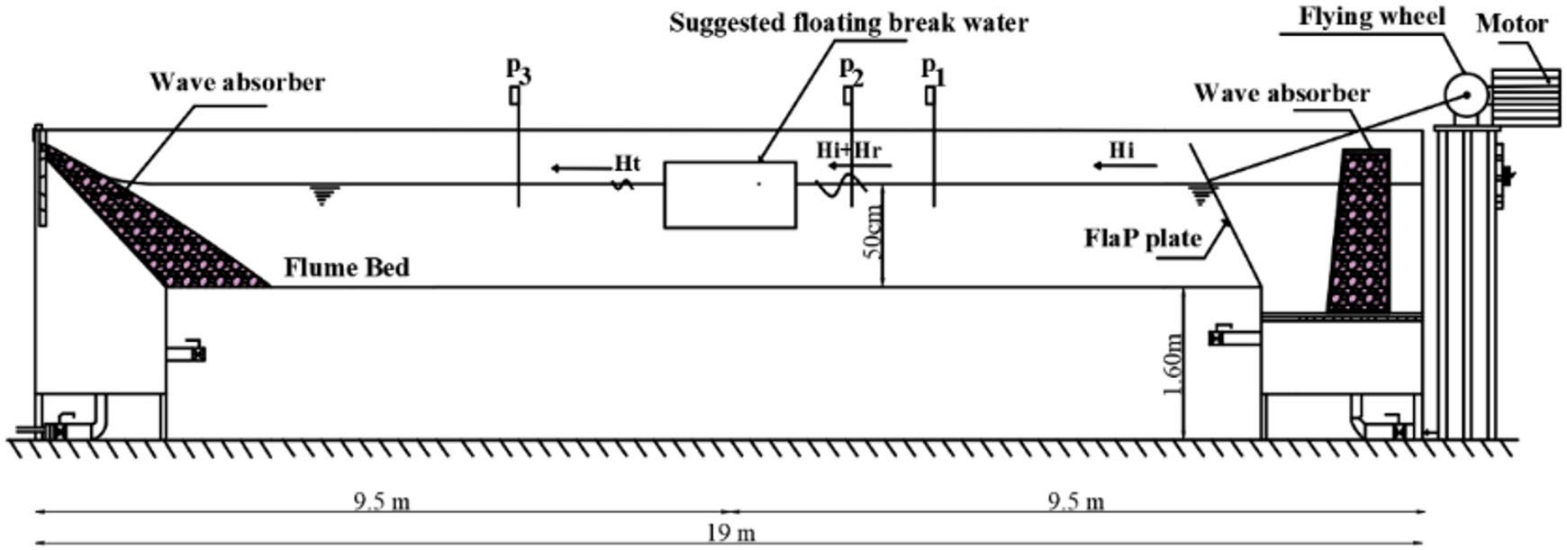
Wave flume description.

The experimental tests were conducted at the Irrigation and Hydraulic Laboratory, Faculty of Engineering, Mansoura University, Egypt. The wave flume has a total length of 19.0 m, with a width and depth of 1.0 m each. The device is equipped with a flap-type wave generator positioned at the upstream end, which generates regular waves that propagate parallel to the longitudinal axis of the flume. Additionally, a gravel-filled screen was placed near the wave maker to minimize wave reflection from the flume walls and the generator itself. The middle part of the wave flume was used for model placement and for the acquisition of experimental measurements. The downstream end of the flume has a wave absorber with a 1:7 slope to absorb the transmitted waves and prevent wave reflection. The channel has a feeding inlet and a drainage valve, which allows accurate adjustment of the water level during testing. Throughout the experiments, the water depth was kept constant at 0.50 m (Fig. 1). To ensure a comprehensive evaluation of the performance of the proposed floating breakwaters, the tested wave periods were 0.6, 0.7, 0.8, 0.9, 1.2, 1.3, 1.5, 1.7, and 2.0 s to cover a wide range of wave conditions.

**Fig. 2:**
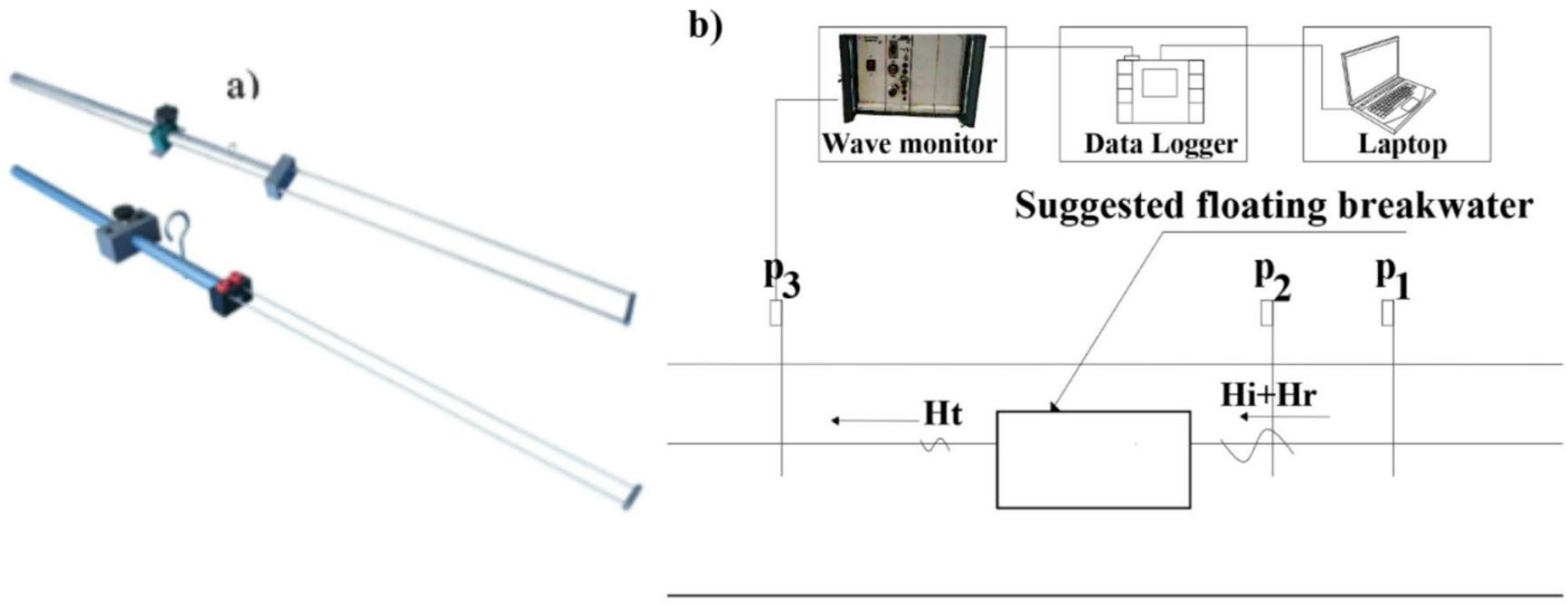
Wave measurement tools: (**a**) Wave probe, and (**b**) Schematic representation of data acquisition framework.

The incident wave heights H_i_ and transmitted wave heights H_t_ were measured by using high-precision capacitance-type wave probe featuring a strict measurement accuracy of ± 0.1 mm and connected to a multichannel data logger (Fig. 2). The probe continuously recorded the instantaneous water surface fluctuations. To ensure high signal sensitivity, the analogue voltage signals were processed via a multi-channel wave monitor at an internal frequency of 50 Hz. To optimize data storage while maintaining signal integrity, the time-series signals were systematically down sampled and digitized through a multi-channel data logger at a recording sampling frequency of 10 Hz (10 stored samples per second). The data logger was interfaced with a laptop, where the acquired time-series signals were automatically transferred and stored using the manufacturer’s software (Fig. 2). To convert the raw voltage signals into physical surface elevations, a linear calibration procedure was performed for the wave probes via incremental immersion tests. The conversion is governed by the following linear relationship:

1 $$\:\eta\:\left(t\right)=\mathrm{C}\cdot\:\mathrm{V}\left(\mathrm{t}\right)+\mathrm{b}$$

where $\:\eta\:$_(t)_ :represents the instantaneous water surface elevation, V _(t)_ : is the measured voltage output, C: is the calibration constant (slope), b: is the intercept.

The range of validity for the wave probe calibration was established between + 15 cm and − 15.0 cm relative to the static water level, which safely encompasses the maximum potential wave crests and troughs generated during the testing matrix. The linear calibration constants (*C and b*) demonstrated exceptional precision, yielding a coefficient of determination (R^2^) exceeding 0.995, with a maximum calibration uncertainty of less than ± 0.1 mm. The calibrated water surface elevation data were subsequently processed using a dedicated analytical routine to identify wave peaks and troughs, from which the average wave height was computed.

To evaluate the experimental repeatability and quantify measurement uncertainty across the testing configurations, statistical analysis was performed on the triplicate runs. The repeatability of the wave height records was assessed using the Coefficient of Variation (*CV*), defined as the ratio of the standard deviation (σ) to the mean value (µ). Across all experimental configurations, the calculated *CV* for both incident and transmitted wave heights remained consistently below 3.5%, confirming the high stability of the wave generation system and the rigorous reproducibility of the laboratory environment.

Following the data acquisition and calibration protocol, each experimental run was limited to a precise duration of 30–60 s. This short duration was strategically selected as a control mechanism to record a sufficient number of stable wave cycles while completely avoiding any potential data contamination from wave reflections originating at the boundaries of the flume. To guarantee statistical reliability and eliminate experimental anomalies, all test configurations were conducted in triplicate. The steady state, reflection-free portion of the stored time-series data was subsequently isolated, and the final wave parameters were determined by averaging the properties of the stable wave train.

Additionally, scaled grids were mounted along the transparent side of the wave flume to allow high-resolution visual recording of wave heights and wavelengths using a high-resolution video camera. These non-intrusive optical measurements were subsequently cross-checked and compared with the digital time-series obtained from the capacitance-type wave probes to cross-validate data consistency and guarantee maximum experimental accuracy.

To ensure wave measurements accuracy, the incident wave heights (H_i_) were initially verified during the flume calibration phase across multiple locations in the empty flume via integrated digital and optical measurement methods, following procedures reported in previous experimental studies (e.g^4,32,33^. Also, after placing the FBW model, the incident wave heights were determined optically at the corresponding upstream locations and computed using the method of Goda and Suzuki^34^, and the calculated magnitude agreed quite closely with the measured values. Following the installation of the FBW model, the transmitted wave heights (H_t_) were systematically recorded at a downstream distance of 1.5 m to isolate the measurements from any localized turbulence caused by wave overtopping. The locations of wave probes are illustrated in Fig. 1.

### Physical modeling scale

The Froude similarity was adopted in this experimental study to ensure accurate simulation of wave–structure interactions. Therefore, a geometric scale of 1:30 was chosen to define the model dimensions and wave characteristics. The proposed breakwater is intended for real-world applications at water depths ranging from 10 to 15 m. Therefore, all the laboratory tests were carried out at a constant water depth of 0.50 m, with wave periods varying from 0.6 to 2.0 s. These values correspond, at the prototype scale, to a water depth of approximately 15 m and wave periods ranging between 3.3 and 10.9 s. While Froude similitude governs the dominant gravity-driven wave dynamics and is the standard requirement for wave–structure interaction models, it does not simultaneously preserve the Reynolds number. However, in floating breakwater experiments where inertial forces dominate over viscous forces, the Froude number takes precedence over the Reynolds number^35^. Furthermore, numerical simulations of wave–structure interaction have shown that the viscous shear force component in the wave propagation direction is less than 0.1% of the total force, confirming that scaling errors due to viscous effects are negligible^36^. The adopted scale of 1:30 also falls within the typical range of 1:30 to 1:50 recommended for breakwater stability and wave–structure interaction studies^37^. Nevertheless, a residual uncertainty associated with Reynolds-scale effects cannot be entirely excluded, as the viscous force component remains unscaled in Froude models, and this is acknowledged as a limitation of the present study.

### Experimental model configurations

**Fig. 3:**
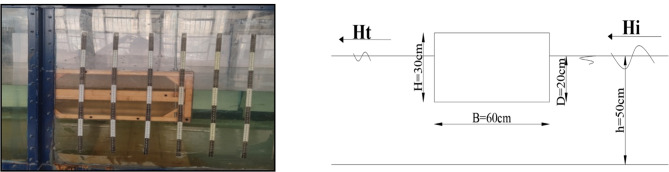
The first configuration model consisted of a standalone rectangular unit.

**Fig. 4:**
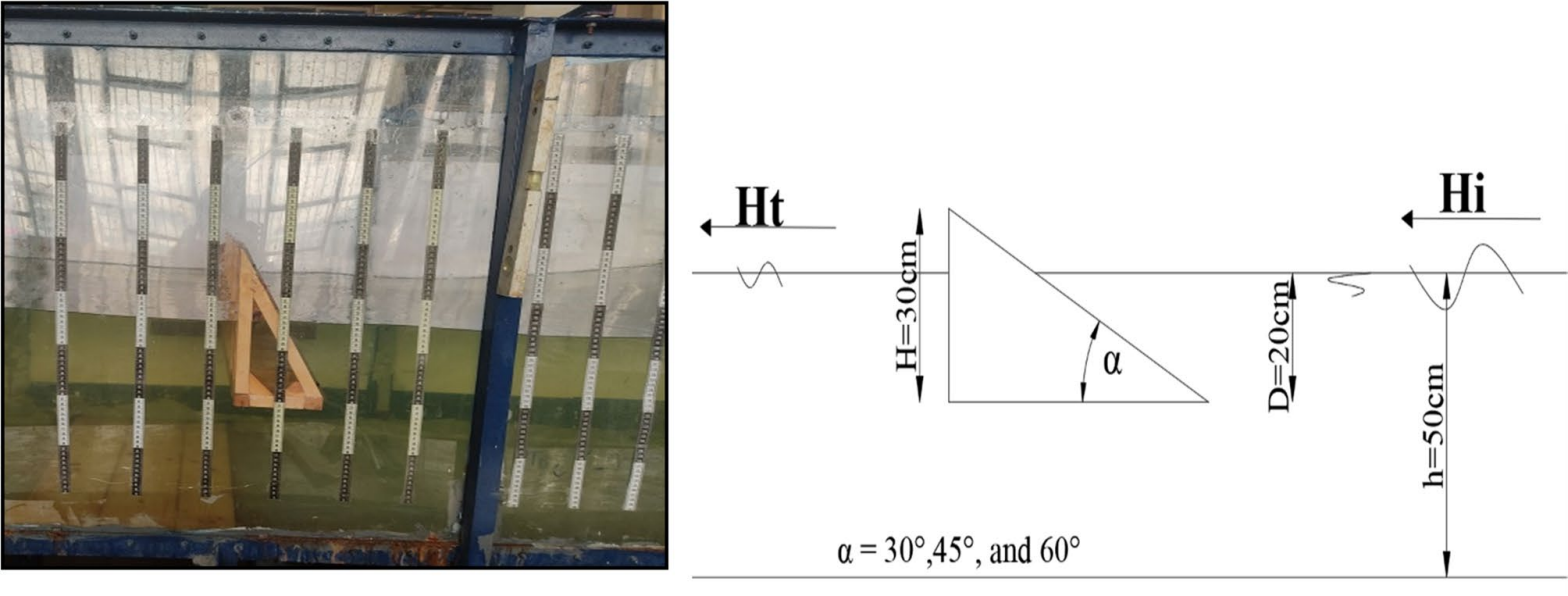
The triangular FBW.

Multiple model configurations were investigated to evaluate the performance of rectangular floating breakwater and the impact of shape modifications. The tested models were oriented perpendicular to the wave direction to ensure proper interaction between the incoming waves and the tested models. The primary configuration consisted of a standalone rectangular unit with dimensions of 100 cm in length, 60 cm in width and 20 cm in draught and a total height of 30 cm (comprising a 20 cm draught and a 10 cm freeboard), as shown in Fig. 3. Subsequently, a second series of tests was conducted using three upright triangular modules to evaluate the attenuation capacities of the individual waves. The triangular modules have base angles of 30°, 45°, and 60° and maintain the same total height as the rectangular FBW (Fig. 4). The selection of the inclination angles of 30°, 45°, and 60° was governed by both physical boundary conditions and practical constructability. To evaluate a comprehensive spectrum of structural slopes between a flat vertical wall (α = 90^o^ ) and a horizontal plane (α = 0^o^ ), three standard engineering slopes were established. An angle of 45° represents a moderate 1:1 slope profile, whereas 60° and 30°configurations simulate steep and gentle slope bounds, respectively, ensuring that the tested profiles remain highly replicable for prototype coastal structures.

**Fig. 5:**
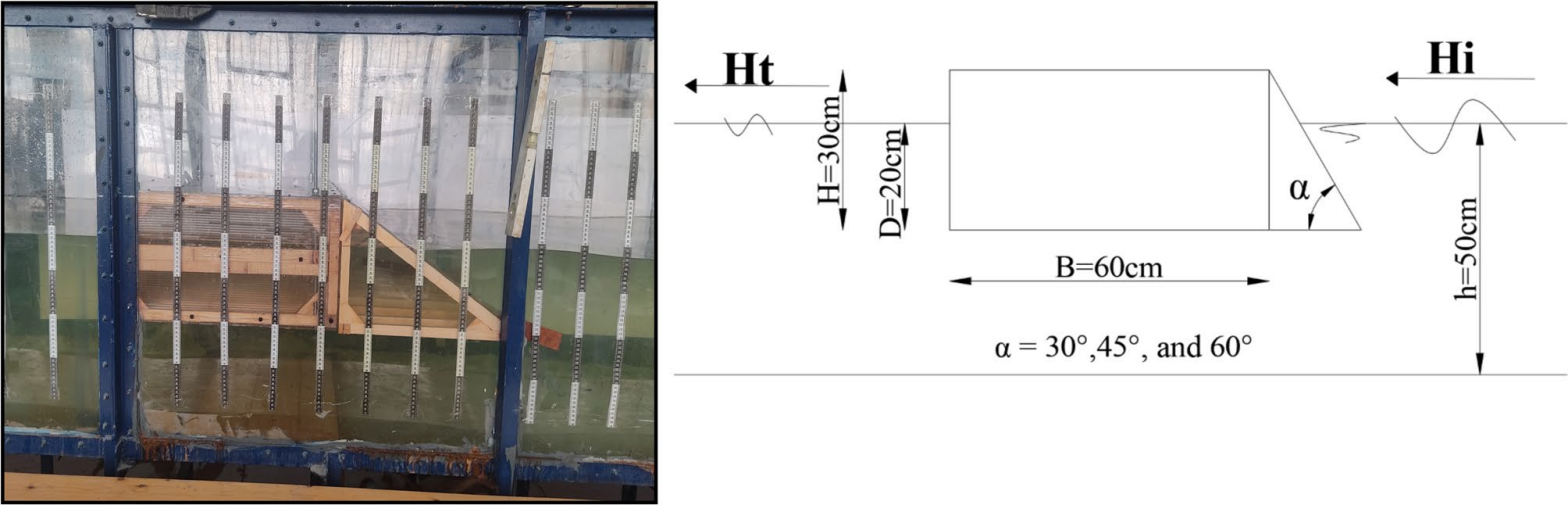
The rectangular FBW with upright triangular module at seaward side.

**Fig. 6:**
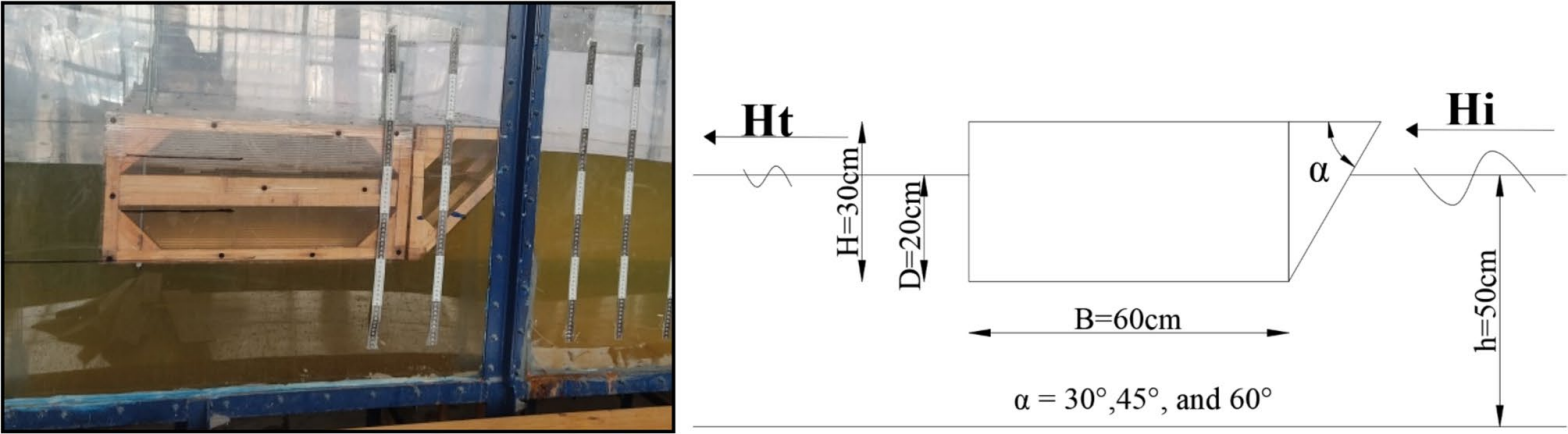
The triangular module in an inverted position, in front of the rectangular FBW.

Then, to examine the trapezoidal shape, the rectangular unit was integrated with the three upright triangular modules placed on the seaward side of the FBW (Fig. 5). Furthermore, a fourth configuration (Model 4) involved orienting each triangular module in an inverted position in front of the rectangular unit to evaluate the impact of the inclined surface on wave energy dissipation (Fig. 6). To isolate the hydrodynamic effects of slope angles, orientations, and configurations on wave transmission, other dimensional parameters including the breakwater draft, freeboard, and total width were kept constant throughout the experimental matrix to avoid parameter cross-interference.

**Fig. 7:**
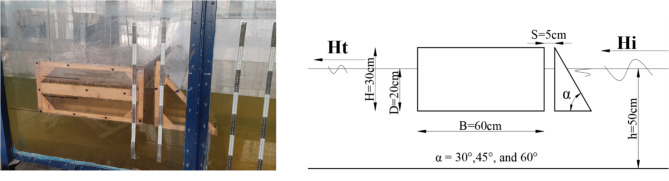
Upright triangular module in front of the rectangular FBW with a 5 cm gap.

**Fig. 8:**
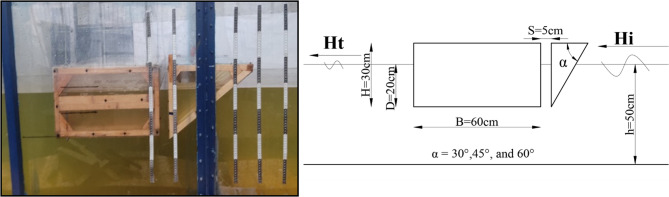
Inverted triangular module in front of the rectangular FBW with 5 cm spacing.

Moreover, in the fifth configuration, the upright triangular modules were placed seaward of the rectangular FBW with a consistent 5 cm gap between the units (Fig. 7). The clear spacing between the modular elements was fixed at 5 cm, which strictly corresponds to 10% of the experimental water depth (h = 50 cm). At the selected model scale of 1:30, utilizing a spacing smaller than 5 cm would trigger restrictive laboratory scale effects where the narrow gap’s hydrodynamic influence becomes negligible and fails to manifest in the flume. Conversely, exceeding a 5 cm spacing represent a prototype distance greater than 1.5 m, changing the hydrodynamic mechanism from a single unified breakwater to double breakwaters with different shapes. Subsequently, the same triangular modules were investigated in an inverted orientation, maintaining the same 5 cm spacing relative to the rectangular unit (Fig. 8). This specific spacing was maintained to evaluate the hydrodynamic interference between the two components and its subsequent effect on wave energy dissipation.

**Fig. 9:**
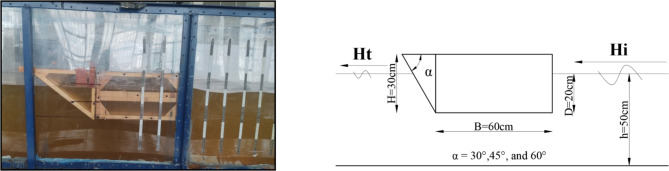
Inverted 30° triangular module behind the rectangular FBW.

**Fig. 10:**
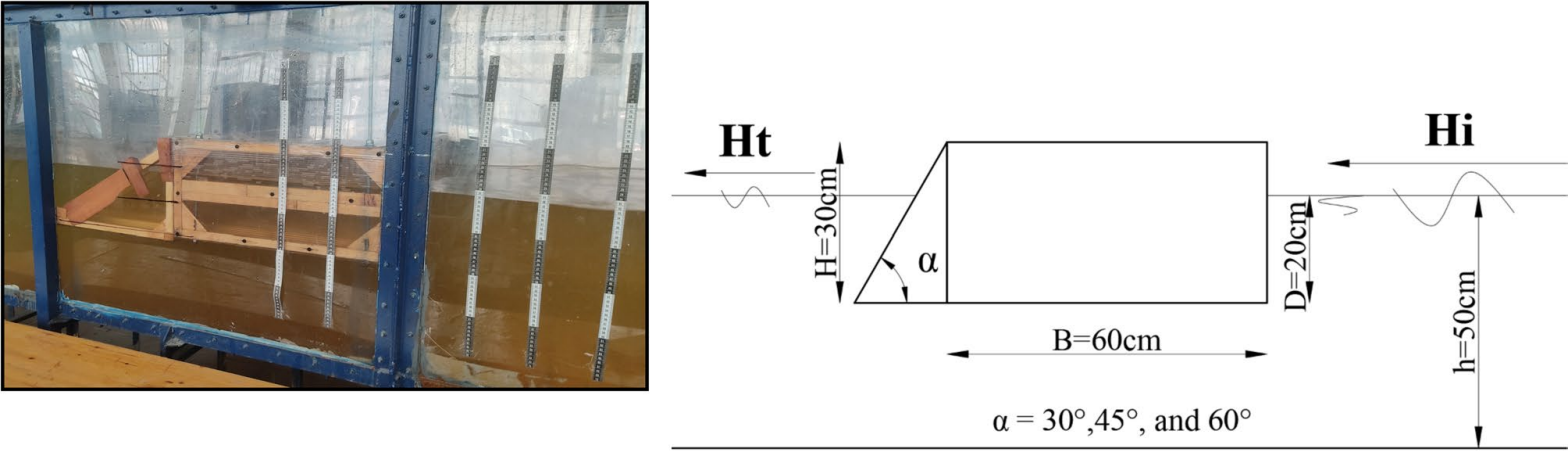
Upright triangular module located behind the rectangular FBW.

Thereafter, Model 7 involved positioning a single 30° inverted triangular module leeward of the rectangular FBW to assess the influence of placing the inclined surface downstream, as shown in Fig. 9. Then, in Model 8, the upright triangular module was similarly located behind the rectangular FBW to evaluate the combined effects of module orientation and leeward positioning, as shown in Fig. 10. Finally, the latter configuration was re-evaluated using a 5 cm clear spacing between the units to determine the impact of structural separation on hydraulic performance. These various configurations were implemented to systematically investigate the impact of shape modification on the wave attenuation performance of the rectangular FBW.

### Description of wave conditions

Experimental investigations were carried out under the action of regular, normally incident waves. The wave periods (T) ranged from 0.6 to 2.0 s, with corresponding wavelengths (L) varying between 56.2 cm and 405.8 cm. Additionally, the incident wave heights (H_i_) used in the tests ranged from 5.76 cm to 10.75 cm. Table 1 provides a detailed summary of the generated wave conditions and the experimental configuration of the tested FBW models.

**Table 1:** Experimental configurations of the proposed breakwater system.

Parameter category	Experimental parameter	Range
Wave parameters	Water depth(h)	50.0 cm
	Wave Period (T)	0.6 to 2.0 s
	Wave Length(L)	56.2 to 405.8 cm
	Incident wave height (H_i_)	5.76 to 10.75 cm
	Wave steepness(H_i_/L)	0.0142 to 0.1913
Model parameters	Breakwater length (l)	100 cm (perpendicular to the wave direction)
	Breakwater draft (D)	20.0 cm
	Breakwater freeboard (f)	10.0 cm
	Breakwater height (H)	30.0 cm
	Used inclination angles (α)	30°, 45°, and 60°
Material & Mooring	Model Material	5 mm thick transparent acrylic sheets
	Mooring System	Fixed

To ensure scalability, adherence to coastal engineering best practices, and to facilitate direct comparisons with international benchmarks, all primary geometric and hydrodynamic variables were transformed into dimensionless parameters. The structural baseline configuration is characterized by a constant relative draft (D/h = 0.40), a relative freeboard (f/h = 0.20), and a relative breakwater height (H/h = 0.60). Throughout the experimental program, the dynamic hydrodynamic boundaries spanned a wave steepness range (Hi/L) from 0.0142 to 0.1913, and a relative breakwater width (B/L) ranging from 0.15 to 1.07. To maintain strict control over the experimental variables, all tests were conducted under controlled laboratory conditions. The physical models were rigidly fixed within the flume to isolate the direct influence of cross-sectional geometry and face orientation from the complex coupled motions of mooring systems. Testing was limited to regular wave configurations at a constant water depth (h = 50 cm), dictated by the physical generation capabilities of the laboratory flume. Furthermore, to minimize laboratory scale constraints and side-wall reflection effects, all wave profile measurements were systematically captured strictly along the flume’s centerline.

## Experimental results

To assess the model’s efficiency in dissipating wave energy under different wave conditions, both the wave transmission coefficient (*K*_*t*_) and wave steepness (H_i_/L) were calculated as follows:

2 $$K_{t} = {\mathrm{H}}_{{\mathrm{t}}} /{\mathrm{H}}_{{\mathrm{i}}}$$

3 $${\mathrm{Wave}}\;{\mathrm{steepness}} = H_{i} /L$$

where *H*_*t*_: The transmitted wave height, *H*_*i*_: The incident wave height, L: The corresponding wavelength.

Then, the hydrodynamic performance of the FBWs was assessed by plotting the wave transmission coefficient (*K*_*t*_) against the wave steepness (H_i_/L). This approach provides a clear visualization of the impact of wave shape variations on energy transmission. The plotted data exhibited consistent trends across all the tested configurations.

### Hydrodynamic performance of the rectangular FBW

**Fig. 11:**
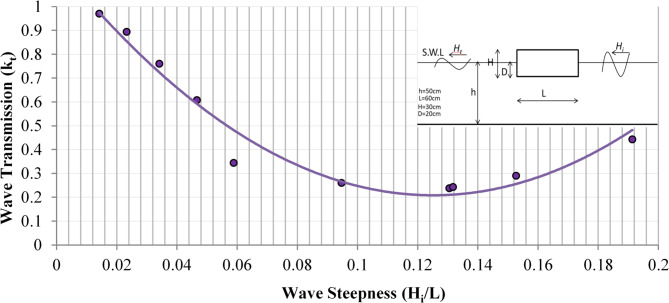
Variation of wave transmission coefficient (K_t_) as a function of incident wave steepness (H_i_/L) for the baseline rectangular FBW configuration.

The hydrodynamic behavior of the rectangular FBW is evaluated by plotting the wave transmission coefficient (*K*_*t*_*)* against the wave steepness (*H*_*i*_/*L)*. As shown in Fig. 11, the initial *K*_*t*_ is high under low wave steepness, with an approximate value of 0.97 at *H*_*i*_/*L* = 0.015, indicating poor wave attenuation due to limited wave-structure interactions. However, as the wave steepness increases, the transmission coefficient gradually decreases, reflecting the improved performance of the breakwater in reducing wave energy. The minimum *K*_*t*_ value was 0.24 at *H*_*i*_/*L* = 0.13, which represents the optimal condition for energy dissipation by the rectangular unit, with an efficiency of 76.0%. Beyond this point, *K*_*t*_ increases again with increasing wave steepness, reaching approximately 0.44 at *H*_*i*_/*L* = 0.19. This upward trend indicates a decrease in performance at high wave steepness, which may be attributed to overtopping over the breakwater crest that was visually observed during the experimental runs.

Therefore, the efficiency of the rectangular FBW increases steadily from approximately 3.0% to 76%, with an average efficiency of 47%, and reaches its peak at *H*_*i*_/*L* = 0.13. Additionally, the rectangular FBW demonstrates effective performance within a mid-range wave steepness interval (approximately *H*_*i*_/*L* = 0.05 to 0.13). However, its efficiency decreases at both very low and very high wave steepnesses, likely due to limited interaction and nonlinear wave effects, respectively.

### Hydrodynamic performance of triangular FBWs

**Fig. 12:**
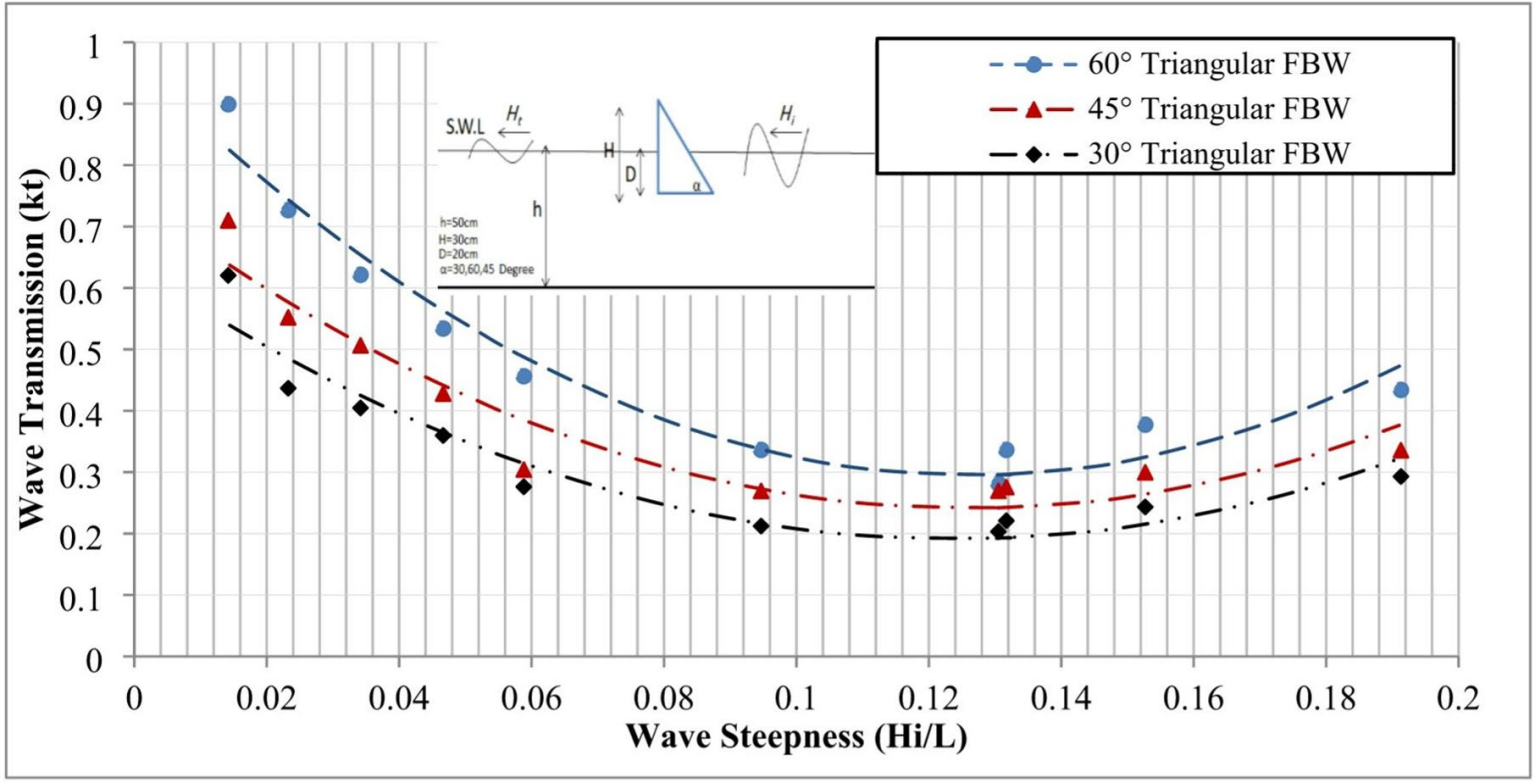
Variation of wave transmission coefficient (K_t_) as a function of incident wave steepness (H_i_/L) for triangular FBW configurations at different base angles (30°, 45°, and 60°).

Figure 12 illustrates the hydrodynamic performance of the triangular FBW on wave transmission. As shown in the figure, all three configurations exhibited similar trends, where *K*_*t*_ decreased progressively as the wave steepness increased until it reached a minimum value, after which it started to rise again. This parabolic behavior indicates that wave attenuation improves up to an optimal steepness value, beyond which the effectiveness of the breakwater decreases. This reduction is attributed to wave overtopping and a decrease in the energy dissipation capacity under high-energy conditions. The figure shows that the 60° triangle consistently shows the highest transmission values across the entire steepness range. The results show that *K*_*t*_ decreases from 0.89 to 0.28 as the wave steepness decreases from 0.015 to 0.13. However, *K*_*t*_ increases again to reach 0.47 as the wave steepness increases to 0.19. In terms of efficiency, the efficiency of the 60° triangle increases from 10% to 72%, with an average value of approximately 48%. Moreover, the 45° triangle performs better than the 60° triangle model when *K*_*t*_ starts at 0.71, and the lowest recorded *K*_*t*_ is approximately 0.27 at *H*_*i*_/*L* = 0.13. Therefore, its efficiency increases from 29% to 73%, with an average value of 59%. Furthermore, the 30° triangle demonstrates the best performance among the three tested triangles, maintaining the lowest *K*_*t*_ values across the entire wave steepness range. As shown, at low steepness, *K*_*t*_ is approximately 0.62, and it drops to a minimum of approximately 0.2 at *H*_*i*_/*L* = 0.13 before increasing again to higher steepness values. Its efficiency improved from 38% to 79%, with an average of approximately 66%.

Hence, as the base angle becomes sharper, the ability of the structure to reflect incoming waves and generate localized turbulence improves significantly. Therefore, acute-angled modules such as the 30° module present a steeper and more abrupt surface to the incident waves, promoting stronger wave reflection and disrupting the flow with higher turbulence intensity. Consequently, a larger portion of the wave energy is dissipated before it can propagate past the structure. In contrast, larger base angles, such as 60°, allow waves to slide along the surface rather than being effectively reflected or dissipated, resulting in higher *K*_*t*_ values and lower overall energy dissipation. Moreover, the observed trend demonstrates that reducing the base angle of the front module leads to a notable improvement in hydrodynamic performance, particularly within the moderate wave steepness range (*H*_*i*_/*L* = 0.05–0.13). However, beyond this range, all the configurations exhibited a decrease in efficiency.

### Comparison between the rectangular FBW and triangular modules

**Fig. 13:**
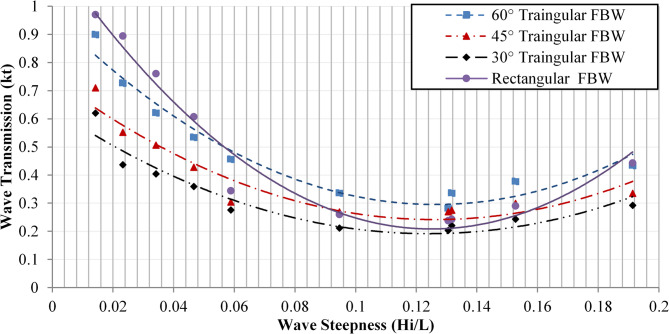
Comparative analysis of wave transmission coefficient (K_t_) versus wave steepness (H_i_/L) between the baseline rectangular FBW and standalone triangular FBW configurations featuring diverse base angles )30°, 45°, and 60°(.

Figure 13 presents a comparative analysis between the triangular FBWs and the rectangular FBW. The results indicate that the rectangular configuration achieves an average efficiency of 47%, while the 60° triangular FBW records a nearly identical average efficiency of 48%, indicating that both configurations exhibit effectively equivalent performance. Additionally, the 45° triangular FBW demonstrates a higher efficiency of 59%, which is approximately 12% greater than that of the rectangular FBW. Moreover, the 30° triangular module exhibits the best performance overall, achieving an average efficiency of 66%, which is approximately 19% higher than that of the rectangular FBW. In addition to hydraulic benefits, the triangular FBWs offer a significant reduction in material requirements. For example, the cross-sectional area of a 30° triangular module is only 44% of that of a rectangular FBW, suggesting a potential cost reduction of approximately 56% in construction materials.

To evaluate the global performance and investigate the statistical significance of the differences among the structural configurations, a comprehensive quantitative comparison was conducted as summarized in Table 2. The analysis reveals that the 30° triangular FBW represents the optimum design alternative, yielding the lowest overall mean transmission coefficient ( µK_t_ = 0.3221) and achieving a peak wave attenuation efficiency of 79.67% (K_t, min_ = 0.2033).

As the frontal inclination angle increases from 30° to 45° and 60°, the mean transmission values increase systematically to 0.4137 and 0.5185, respectively. This drop in performance highlights the hydrodynamic advantage of milder slopes in inducing premature wave breaking and energy dissipation. Crucially, while the conventional Rectangular FBW exhibits a low minimum transmission value under specific steepness conditions, it yields the highest statistical dispersion (σK_t_ = ± 0.2917) and a high overall mean transmission (µK_t_ = 0.5317 ), reflecting a highly unstable performance across the wave spectrum compared to the steady behavior of the triangular modules.

**Table 2:** Quantitative performance, statistical dispersion, and wave attenuation efficiency across the proposed FBW alternatives.

Alternative Configuration	Mean Transmission Coefficient (µK_t_)	Standard Deviation (σK_t_)	Maximum Wave Attenuation Efficiency (η_max_)	Minimum Transmission Coefficient (K_t, min_)
30° triangular FBW	0.3221	± 0.1340	79.67%	0.2033
45° triangular FBW	0.4137	± 0.1540	73.04%	0.2696
60° triangular FBW	0.5185	± 0.1873	71.85%	0.2815
Rectangular	0.5317	± 0.2917	76.15%	0.2385

In conclusion, the triangular FBW configuration provides up to 19% higher efficiency while simultaneously reducing the material volume by more than half. Nonetheless, the practical implementation of these modules must account for specific structural and stability constraints. Hence, to balance performance, cost, and constructability, combining features of both rectangular and triangular configurations may offer an optimal solution.

### Hydrodynamic performance of the trapezoidal FBWs

**Fig. 14:**
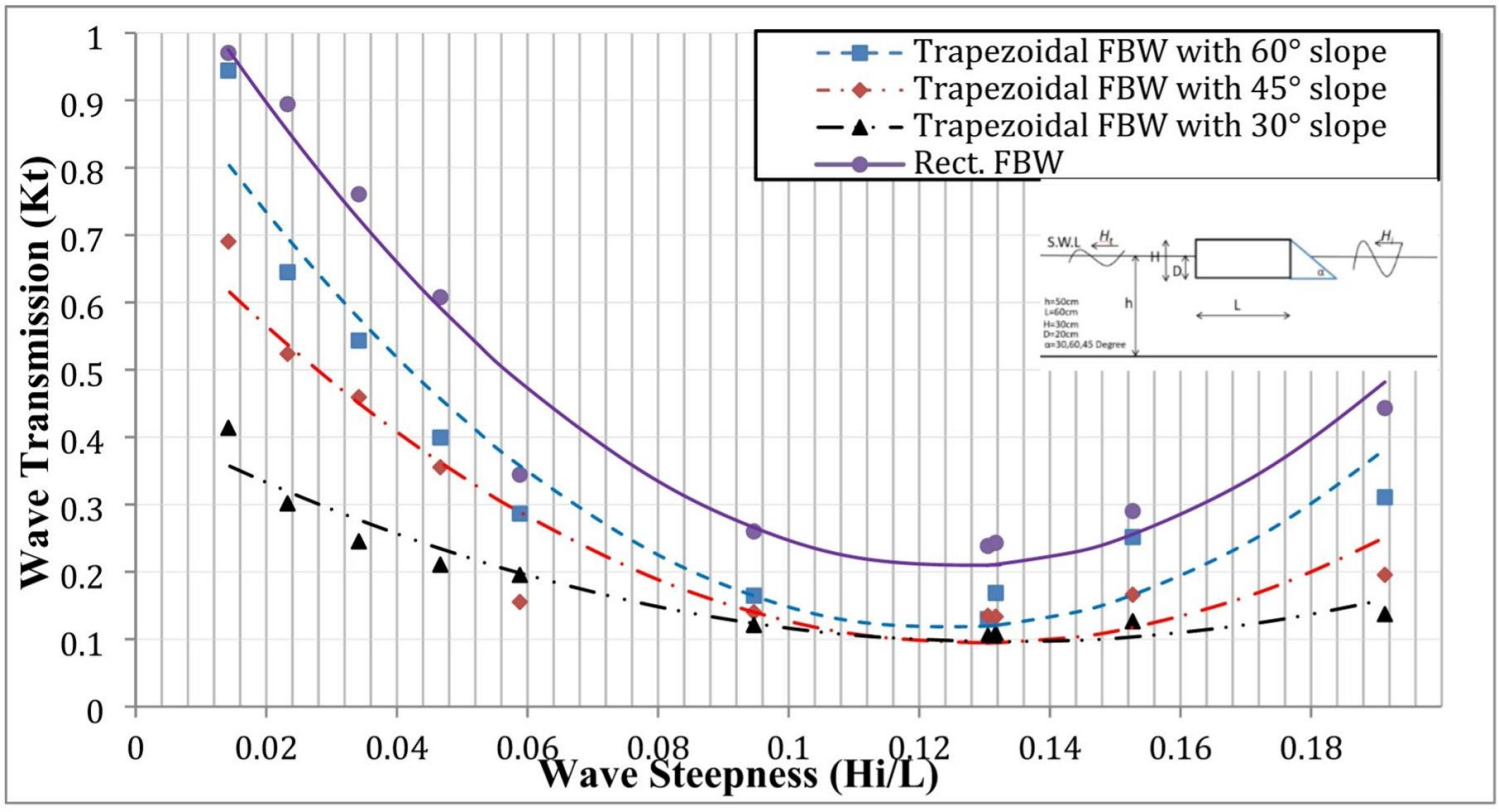
Comparative analysis of wave transmission coefficient (K_t_) against wave steepness (H_i_/L) between the baseline rectangular FBW and upright trapezoidal FBW configurations deployed in the seaward orientation under various base angles )30°, 45°, and 60°(.

To investigate the hydrodynamic influence of a trapezoidal cross-section, composite models were developed by integrating triangular modules with a rectangular FBW. This configuration effectively creates a sloped frontier intended to enhance wave energy dissipation and reduce reflection by modifying the structure’s entry profile. Therefore, the three triangular modules with base angles of 30°, 45°, and 60° were placed ahead of the rectangular FBW. Figure 14 illustrates the impact of changing the base angle of the front slope of trapezoidal FBWs and a comparison between trapezoidal FBWs and the rectangular FBW. In terms of efficiency, the wave attenuation efficiency of trapezoidal FBWs with 60° slopes ranges from 6% to 87%, with an average efficiency of 61.50%, which is approximately 14.5% greater than that of the rectangular configuration. Moreover, the 45° slope performs better than both the rectangular model and the 60° slope. Its efficiency improves from 31% to 87%, with an average of 70%, which is approximately 23% greater than that of the rectangular FBW. However, the 30° slope demonstrates the best performance among all the tested configurations, as the efficiency ranges from 59% to 89%, with an average of 80.30%, which is approximately 33.30% greater than that of the rectangular FBW.

The results clearly indicate that trapezoidal FBWs with sharper base angles significantly improve the wave attenuation efficiency. This superior performance is attributed to the sharp front profile of the 30° triangle, which enhances wave reflection and creates turbulence in front of the structure, leading to greater energy dissipation.

### Hydrodynamic performance of trapezoidal FBWs with an inverted side slope

**Fig. 15:**
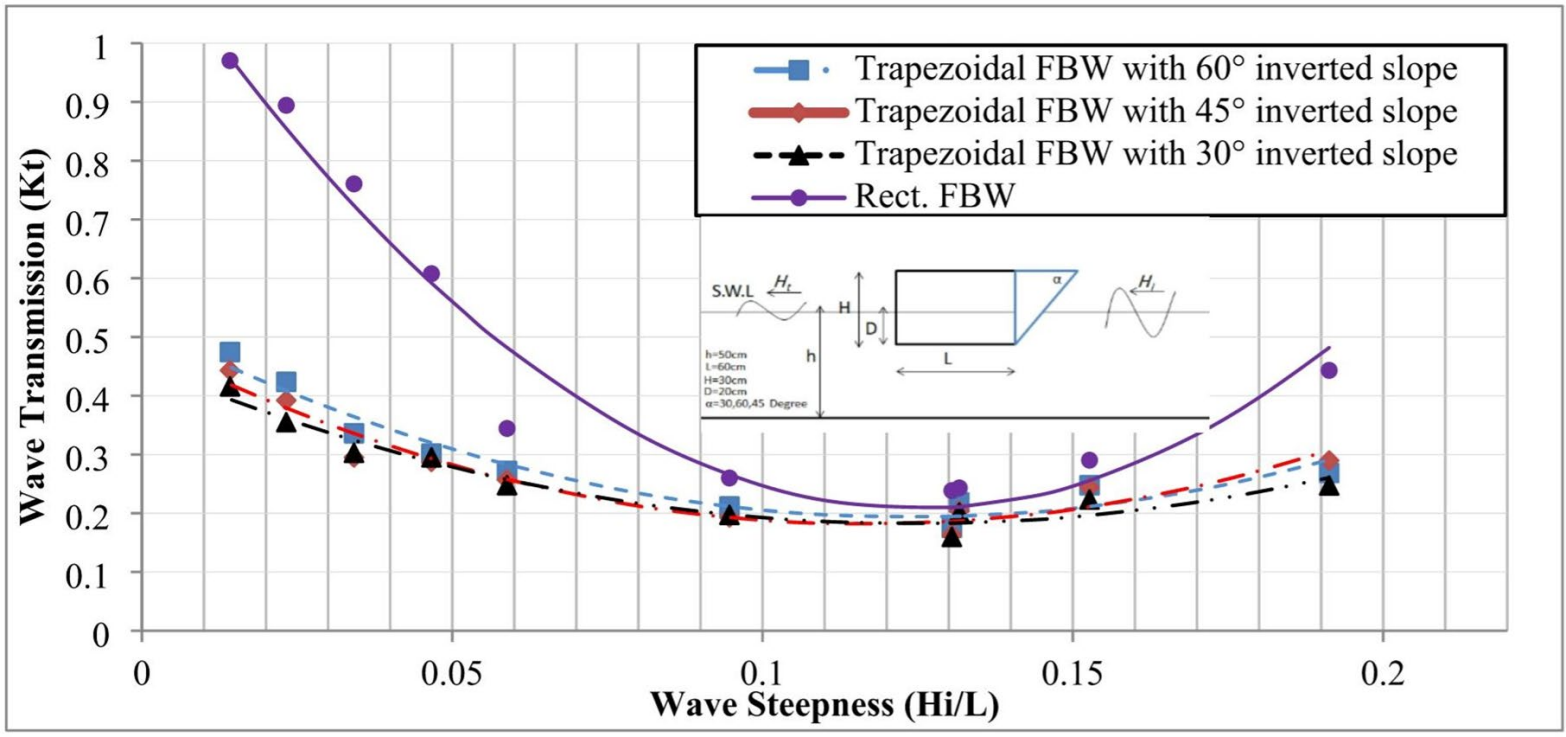
Evaluation of wave transmission coefficient (K_t_) variation against wave steepness (H_i_/L) between the baseline rectangular FBW and trapezoidal FBW configurations with inverted slopes deployed in the seaward orientation under various base angles )30°, 45°, and 60°(.

The triangular modules were placed in front of the rectangular FBW in an inverted position to create a trapezoidal cross-section with an inverted side slope. The results show that the 60° inverted side slope results in a moderate decrease in K_t_, as it ranges from 0.47 to approximately 0.18, before increasing again at higher steepness values (Fig. 15). In terms of hydrodynamic performance, this configuration has a moderate efficiency ranging from 53% to 82%, with an average of 70.7%, which is approximately 23.7% higher than that of the rectangular model. Additionally, when the rectangular FBW is combined with the inverted 45° triangular module, its efficiency ranges from 56% to 84%, with an average of 72.3%, which is 25.3% higher than that of the rectangular FBW. Moreover, the 30° inverted side slope yields the lowest K_t_ starting at 0.42 and reaching a minimum of approximately 0.15 at H_i_/L = 0.13. Its efficiency ranges from 58% to 84%, with an average of 72.5%, which is about 25.5% higher than that of the rectangular model.

Overall, the results clearly indicate that using trapezoidal FBWs with inverted side slopes enhances the wave attenuation efficiency, particularly within a moderate wave steepness interval. However, a change in the triangle angle had an insignificant impact on the transmission coefficient. Therefore, the selection of the most economical option depends on the most practical design choice. These findings emphasize that while geometry affects hydrodynamic performance, economic considerations can guide the optimal choice among comparable configurations.

**Fig. 16:**
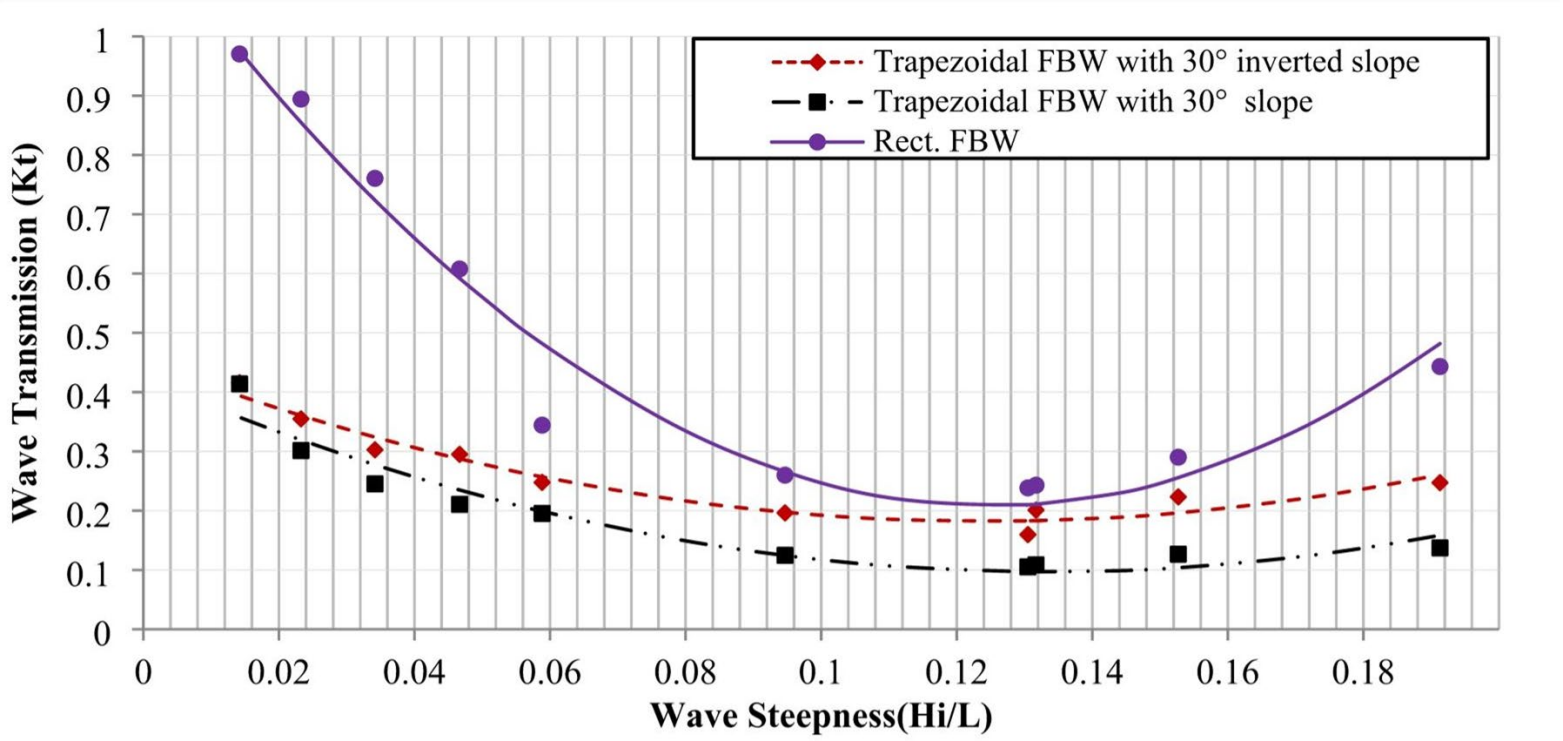
Wave attenuation efficiency (K_t_ versus H_i_/L) comparing the baseline rectangular FBW against trapezoidal FBW configurations at a fixed 30° angle, contrasting the upright and inverted slope configurations in the seaward orientation.

However, the orientation of the triangle used seems to have a significant impact on the hydrodynamic performance and inherent hydrostatic stability of the FBW. Therefore, a comparison between upright and inverted side slopes was used to determine the optimal orientation. As shown in Fig. 16, the use of the upright 30° side slope has a reasonable efficiency, with an average of 80.3%, representing an improvement of about 33.3% compared to that of the rectangular FBW. In contrast, using an inverted 30° side slope resulted in an average efficiency of 73.55%. These results clearly indicate that the upright 30° side slope provides the most effective enhancement. This outcome can be attributed to its sharper and more abrupt leading profile, which enhances wave reflection and intensifies turbulence, thereby increasing energy dissipation relative to the inverted configuration. Therefore, a trapezoidal cross-section with an upright 30° side slope orientation emerges as the most efficient design among the tested cases, offering superior hydrodynamic performance and highlighting the importance of geometric optimization in floating breakwater systems.

### Assessment of the impact of module spacing on trapezoidal FBWs

To investigate the influence of module spacing and hydrodynamic interference, a split trapezoidal configuration was tested. In this setup, the triangular modules were positioned seaward of the rectangular FBW and separated by a clear spacing of 5 cm (S = 5 cm). This dual-element system was evaluated to determine how the clear spacing affects the overall wave transmission and hydrodynamic performance compared to the continuous, gapless configuration.

**Fig. 17:**
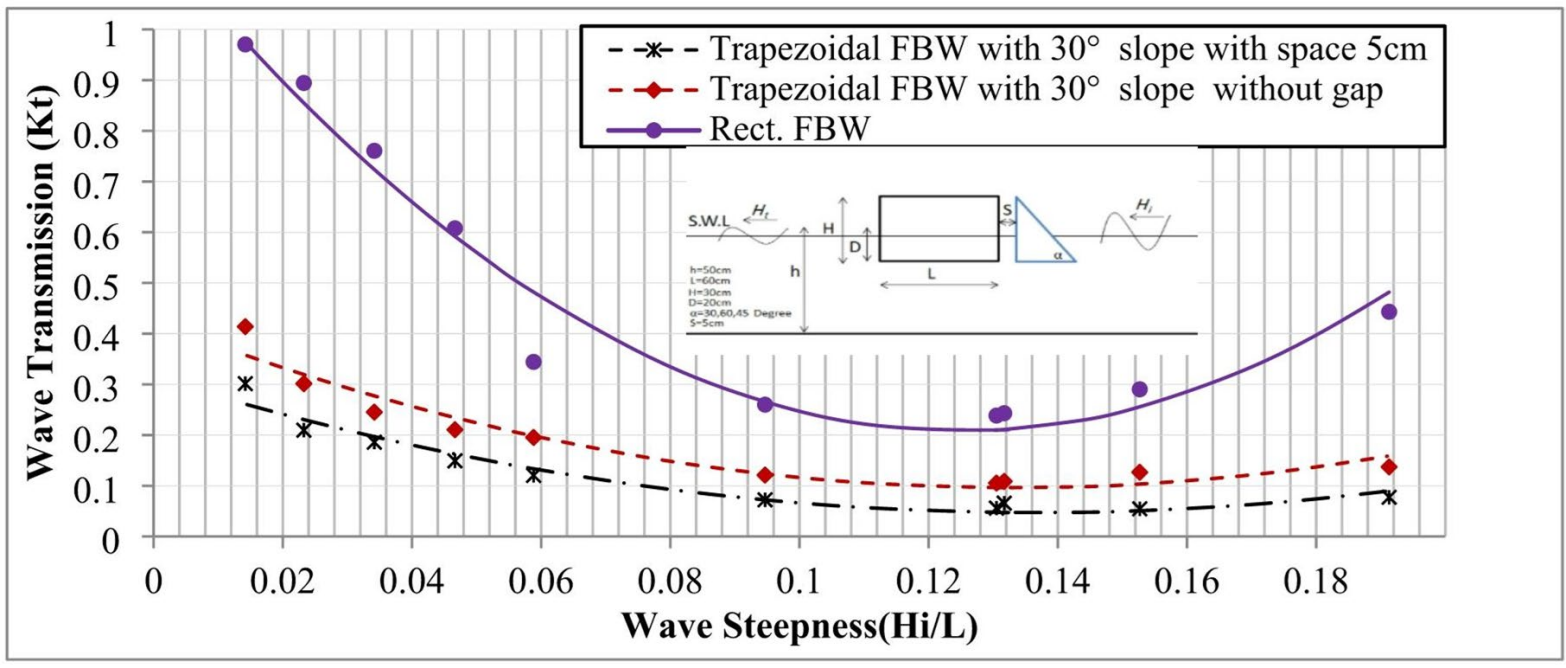
Sensitivity of wave transmission coefficient (Kt) to a 5 cm clear spacing for a 30° upright trapezoidal FBW configuration, contrasting split and contiguous solid structures against the baseline rectangular unit across varying wave steepnesses (Hi/L).

The results show that a noticeable improvement is observed due to the use of space between the models (Fig. 17). The figure shows that the configuration used with a 5 cm gap demonstrated the best performance, with minimum K_t_ values ranging from 0.30 to 0.06 at H_i_/L = 0.13. So, the tested model efficiency ranges from 69% to 94%, with an average of 87%, which is nearly 40% higher than that of the rectangular model. In contrast, the 30° triangular module without spacing achieves an average efficiency of 80.3%, confirming that the spacing enhances the overall performance. However, increasing the space may have significant drawbacks for breakwater stability.

The observed improvement in wave attenuation with 5 cm spacing in upright configurations can be attributed to the beneficial “buffer zone” created by the gap. This space allows for the partial dissipation of wave energy between the triangle and the main structure before the waves encounter the rectangular unit. The interaction within this cavity enhances turbulence and contributes to a gradual reduction in wave energy, resulting in a cumulative damping effect. These findings clearly demonstrate that optimizing the shape and placement of structural modules is essential for improving the efficiency of floating breakwaters across a range of wave environments.

### Comparative assessment of side slope location relative to incident waves

To evaluate the influence of slope orientation relative to incident waves, a comparative analysis was performed using two trapezoidal cross-sections. Both configurations featured a 30° side slope; however, the slope was positioned seaward in the first model and leeward (landward) in the second. This comparison aims to determine which orientation provides superior wave energy dissipation and lower transmission coefficients. Furthermore, to investigate the influence of structural separation, these configurations were re-evaluated by introducing a 5 cm clear spacing between the triangular and rectangular components. This test aims to determine whether a contiguous trapezoidal profile or a dual-body system with an internal damping chamber provides superior wave energy dissipation.

**Fig. 18:**
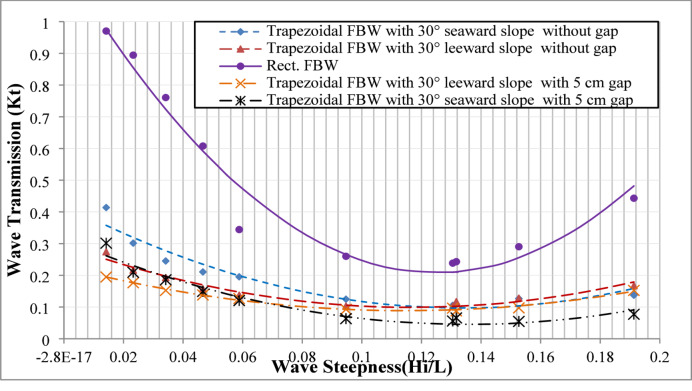
Investigation of wave attenuation variance (K_t_ vs. H_i_/L) under parametric variations of slope placement (seaward vs. leeward orientations) and structural integration (with 5 cm gap vs. without gap) for a 30° trapezoidal FBW, contrasted with the baseline rectangular unit.

The results show that the 30° slope on the leeward side provides a reasonable transmission coefficient of 0.27 at low steepness, which decreases to 0.10 at H*i*/L = 0.13 (Fig. 18). This configuration provides high wave attenuation, with a performance ranging from 73% to 89%, with an average of 83%. Conversely, when the 30° triangular module is placed on the seaward side, the overall efficiency reaches 79% on average.

Therefore, these results indicate that both trapezoidal arrangements provide better performance than rectangular breakwater. However, positioning the 30° side slope on the leeward side yields better hydrodynamic efficiency. This improvement can be explained by the fact that the rectangular body first dissipates the majority of the incoming wave energy, while the rear-positioned triangular module interacts primarily with the residual wave motion. This secondary interaction enhances turbulence and reflection behind the main structure, resulting in an additional layer of energy dissipation. Overall, the findings confirm that the location of the triangular module plays a critical role in wave attenuation and that the rear configuration provides the most effective energy dissipation.

Moreover, a minor improvement is observed due to using space on the leeward side, as the figure shows K_t_ values ranging from 0.19 to 0.09. Therefore, its efficiency ranges from 80% to 91%, with an average of 86.8%. Notably, the seaward spacing outperformed the leeward spacing, exhibiting the highest overall performance, with an efficiency ranging from 69% to 94% and an average of 87%. Hence, the analysis revealed that the leeward gap had an insignificant impact, as it increased the average efficiency by approximately 2.8%. Additionally, using the spacing on the seaward side enhances the overall efficiency of the test model by approximately 6.8%. However, considering inherent hydrostatic stability and ease of fabrication, the contiguous trapezoidal configuration (without a gap) is recommended for practical implementation.

**Fig. 19:**
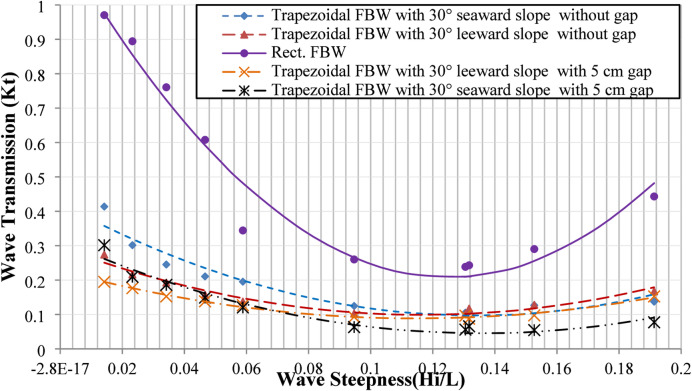
Performance optimization analysis (K_t_ vs. H_i_/L) comparing 30° trapezoidal FBW configurations with inverted slopes (deployed in seaward and leeward orientations) against the optimized upright leeward slope configuration and the baseline rectangular unit.

Finally, to optimize performance and sustain a broad view, the inverted 30° triangular module was placed on the leeward side to assess whether it delivered the same level of performance. The findings reveal that K_t_ ranges from approximately 0.36 to 0.12 at H_i_/L = 0.13. Moreover, the configuration performance ranged from 64% to 85%, with an average of 77%, which is approximately 5% greater than that of using the same module on the seaward side (Fig. 19).

In order to establish a comprehensive architectural benchmark, Table 3 delivers a direct cross-comparative analysis between the optimum configurations of the evaluated breakwater typologies: the 30° triangular, the trapezoidal (with 30° leeward slope), and the Conventional rectangular FBW. The quantitative indicators demonstrate that the trapezoidal FBW outperforms all other cross-sections significantly, achieving an exceptional overall mean transmission coefficient of just (µK_t_ = 0.1706) and a peak wave attenuation efficiency of 89.75%. Physically, this supreme performance is attributed to the combined geometric advantage of the trapezoidal section; the front and leeward slopes work in tandem to maximize energy dissipation via controlled wave overtopping and turbulence generation, while its broader top width enhances the reflection dampening.

Furthermore, the Trapezoidal FBW exhibits the highest structural stability across the entire wave spectrum, as mathematically validated by its remarkably narrow standard deviation ( σK_t_ = ± 0.0543). In comparison, while the triangular section performs steadily, the rectangular section yields a highly chaotic and inefficient performance profile, cementing the necessity of implementing sloped or trapezoidal geometries in modern coastal protection layouts.

**Table 3:** Comparative cross-sectional performance and efficiency matrix across different FBW typologies.

Alternative configuration	Mean Transmission Coefficient (µK_t_)	Standard Deviation (σK_t_)	Maximum Wave Attenuation Efficiency (η_max_)	Minimum Transmission Coefficient (K_t, min_)
Rectangular	0.5317	± 0.2917	76.15%	0.2385
30° triangular FBW	0.3221	± 0.1340	79.67%	0.2033
Optimum Trapezoidal FBW (with 30° Leeward slope)	0.1706	± 0.0543	89.75%	0.1025

Hence, the findings confirm that the trapezoidal FBW exhibits superior hydrodynamic performance when the sloped face is positioned on the leeward side rather than the seaward side. In this configuration, the vertical seaward face acts as the primary barrier to reflecting incident wave energy, while the subsequent leeward slope facilitates a smoother transition of the remaining flow, significantly reducing the transmission coefficient. Furthermore, the upright orientation of the slope was found to be more effective than the inverted orientation, particularly when the slope was situated on the leeward side. This suggests that a leeward, upright trapezoidal profile represents the most efficient geometry for minimizing wave transmission and maximizing energy dissipation.

## Discussion

The hydrodynamic performance of the various floating breakwater (FBW) configurations was evaluated by analyzing the wave transmission coefficient (K_t_) against the wave steepness (H_i_/L). The rectangular FBW exhibited an average efficiency of 47%, with a peak performance of 76% efficiency at a mid-range wave steepness (H_i_/L = 0.13). The efficiency decreased at lower wave steepnesses, which typically correspond to longer wave periods where the relative breakwater width (B/L) is small, allowing the wave energy to pass with minimal structural interaction. Conversely, at higher steepnesses, the performance degradation is governed by non-linear wave overtopping and transmission. Additionally, all the triangular FBW models exhibited a parabolic trend; however, the 30° triangle emerged as the superior standalone geometry. This model achieved an average efficiency of 66%, which is 19% better than that of the rectangular model, while utilizing only 44% of the cross-sectional area of the rectangular unit. This demonstrates that sharper base angles promote stronger wave reflection and localized turbulence, whereas larger angles allow waves to slide along the surface.

The observed 19% improvement in efficiency is fundamentally attributed to the transition from a reflection-dominated mechanism (characteristic of the vertical-faced rectangular FBW) to a dissipation-dominated mechanism (provided by the 30° triangular profile). Unlike the rectangular unit, which reflects a substantial portion of incident wave energy back into the basin, the 30° triangular module introduces a sloped surface that extends the wave run-up and interaction path. Physically, this milder slope forces the incoming wave to undergo a gradual breaking process, which maximizes energy dissipation via intense bottom-boundary friction and organized vortex shedding. Furthermore, the inclination of the face promotes flow separation at the structural edges, transforming potential wave energy into turbulent kinetic energy more efficiently than a vertical face. Consequently, the 30° module does not merely reflect the wave; it actively degrades the wave’s orbital velocity and energy density before it reaches the leeward side of the structure. This transformation shifts the primary hydrodynamic mechanism from pure potential flow reflection to a viscous-dominated dissipation regime.

On the other hand, integrating triangular modules with a rectangular FBW to form a trapezoidal profile significantly enhances wave attenuation. The upright 30° side slope orientation proved most effective, averaging 80.3% efficiency. While inverted slopes also improved the performance over the rectangular baseline to reach 73.5% on average, changes in the base angle had an insignificant impact on K_t_ in the case of the inverted configurations. Additionally, the sharper leading profile of the upright slope intensifies turbulence and reflection more effectively than does the inverted profile.

Moreover, the use of a 5 cm clear spacing between modules created a “buffer zone” that acted as an internal damping chamber, facilitating localized phase-shifting of the wave profile and destructive hydrodynamic interference that promotes gradual energy reduction. Therefore, using a seaward gap for the rectangular unit provided the highest overall performance, with a mean efficiency of 87%.

Furthermore, positioning the 30° upright slope on the leeward side yielded better results than the seaward placement. In the leeward setup, the vertical seaward face acts as the primary reflection barrier, while the rear slope interacts with the residual wave motion to provide a secondary layer of dissipation. Therefore, the leeward configuration achieved an average efficiency ranging from 83% (solid) to 86.8% (with spacing). In contrast, the seaward configuration efficiency ranged from 80.3% (solid) to 87% (with spacing). Finally, the results demonstrate that while the seaward-spaced split trapezoid achieved the maximum numerical efficiency of 87%, the contiguous leeward-sloped trapezoidal profile offers the best balance of high hydrodynamic performance (83%), structural stability, and ease of fabrication.

In summary, the holistic synthesis of these experimental outcomes clearly demonstrates how the overarching research objectives have been systematically realized. The primary mission of this investigation was to evaluate the hydrodynamic and economic viability of altering cross-sectional breakwater geometries, with a particular focus on face dynamics and inclination. As established by the baseline testing, transitioning from the conventional rectangular FBW to the optimized standalone 30° ^t^riangular FBW successfully improved the average wave attenuation efficiency by 19% (rising from 47% to 66%). Crucially, this geometric transition simultaneously achieves a massive 56% reduction in material volume, utilizing only 44% of the rectangular unit’s cross-sectional area, which highlights a profound economic advantage.

To maximize these independent benefits, the trapezoidal profile was engineered as a hybrid evolution—successfully marrying the extended top-width reflection dampening of the rectangular design with the sloped-face turbulence generation of the triangular design. By deploying this hybrid geometry in an upright 30° leeward-sloped configuration, the system achieves a major breakthrough. It yields an average efficiency of 83% in its contiguous solid form, which marks a substantial 36% absolute efficiency increase over the conventional rectangular baseline, and a 17% advancement over the standalone triangular module.

Consequently, these findings validate that strategic modifications to the structural face and cross-sectional typology do not merely offer incremental changes, but rather dictate the overall fluid dissipation mechanism. The contiguous leeward-sloped trapezoidal profile emerges as the definitive optimum alternative, providing the ideal engineering balance of elite hydrodynamic performance, inherent hydrostatic stability, and fabrication feasibility. Also, the present experiments provide useful physical insight into the hydrodynamic performance and attenuation mechanisms of the proposed FBW, but one of the main limitations should be acknowledged that the experiments were conducted in a two-dimensional wave flume under normally incident regular waves.

## Conclusion

This study investigated the hydrodynamic performance of various floating breakwater (FBW) configurations through experimental flume testing. The primary mission of this investigation was to evaluate the hydrodynamic and economic viability of altering cross-sectional breakwater geometries, with a particular focus on face dynamics and inclination. By analyzing the wave transmission coefficient (K_t_) across different geometries and orientations, the following conclusions are drawn:

1. The tested 30° triangular module achieved an average efficiency of 66%, which represents a 19% improvement over the standard rectangular FBW while requiring 56% less material, offering a superior cost-to-performance ratio.
2. Integrating triangular modules with a rectangular core to form a trapezoidal profile significantly enhances wave attenuation.
3. The upright orientation of the side slope is more effective than the inverted orientation, as its sharper leading profile intensifies wave reflection and turbulence.
4. The slope orientation relative to the incident waves is critical; positioning the sloped face on the leeward side provides better performance than does seaward placement, establishing a secondary layer of dynamic energy dissipation.
5. The structural face and cross-sectional typology do not merely offer incremental changes, but rather govern the underlying fluid mechanics, transitioning the attenuation process from potential flow reflection to a viscous-dominated dissipation regime.
6. Geometric optimization successfully develops high-performance floating breakwaters capable of mitigating high-steepness waves without escalating material volumes, proving that smart structural shaping can replace massive spatial deployment.

For practical design implementation, coastal engineers can utilize these findings as guidelines for modular breakwater optimization. Under standard operational wave climates, the contiguous leeward-sloped trapezoidal profile should be prioritized to maximize both geometric energy dissipation and structural durability. Future research should extend this baseline investigation by exploring non-linear and viscous fluid effects through advanced numerical simulations, alongside expanding experimental testing to accommodate prototype-scale models under multi-directional irregular sea conditions.

## Data Availability

All data supporting the findings of this study are available within the paper.

## References

[1] Tharwat, E., Saad, M. E. & Sarhan, T. Assessment the impact of coastal protective structures on Damietta shoreline behaviour. *J. Coastal. Conserv.* **29** (6), 75 (2025).

[2] Yin, Z. et al. Hydrodynamic characteristics of a box-type floating breakwater with restrained piles under regular waves. *Ocean Eng.* **280**10.1016/j.oceaneng.2023.114408 (2023).

[3] Kamphuis, J. W. *Introduction to coastal engineering and management* Vol. 48 (World Scientific, 2020).

[4] Wu, J. P. et al. Estimation of reflected and incident waves for breakwater tests in a 2D wave flume in regular waves. *J. Ocean. Eng. Sci.*10.1016/j.joes.2025.09.005 (2025).

[5] Ezzeldin, M. M., Rageh, O. S. & Saad, M. I. Assessment impact of the Damietta harbour (Egypt) and its deep navigation channel on adjacent shorelines. *J. Integr. Coastal. Zone Manage.* **20** (4), 265–281 (2020).

[6] Buccino, M., Del Vita, I. & Calabrese, M. Engineering Modeling of Wave Transmission of Reef Balls. *J. Waterw. Port Coast. Ocean Eng.* **140** (4). 10.1061/(asce)ww.1943-5460.0000237 (2014).

[7] Nguyen, H. P. et al. Representative transmission coefficient for evaluating the wave attenuation performance of 3d floating breakwaters in regular and irregular waves. *J. Mar. Sci. Eng.* **9** (4). 10.3390/jmse9040388 (2021).

[8] Van der Meer, J. W., Briganti, R., Zanuttigh, B. & Wang, B. Wave transmission and reflection at low-crested structures: Design formulae, oblique wave attack and spectral change. *Coast. Eng.* **52** (10–11), 915–929 (2005).

[9] Wei, W., Li, Z., Jiang, C., Wang, C. & Cao, Z. Effects of mooring configuration on wave attenuation of an arc-plate dual-pontoon floating breakwater. *AIP Advances*, **15**(12). (2025).

[10] Seelig, W. N. *Two-Dimensional Tests of Wave Transmission and Reflection Characteristics of Laboratory Breakwaters.* (1980).

[11] Oumeraci, H. Review and analysis of vertical breakwater failures—lessons learned. *Coast. Eng.* **22** (1–2), 3–29 (1994).

[12] Dean, R. G. & Dalrymple, R. A. *Coastal processes with engineering applications* (Cambridge University Press, 2004).

[13] Park, J. C., Kim, K. H., Wang, J. H. & Zhao, X. L. A multi-domain BEM approach for hydrodynamic analysis of arbitrarily shaped 2D floating breakwaters with wide porous media. *Ocean Eng.* **343**, 123570 (2026).

[14] Sannasiraj, S. A., Sundar, V. & Sundaravadivelu, R. Mooring forces and motion responses of pontoon-type floating breakwaters. *Ocean Eng.* **25** (1), 27–48 (1998).

[15] Moghim, M. N. & Botshekan, M. Analysis of the performance of pontoon-type floating breakwaters. *HKIE Trans. Hong Kong Institution Eng.* **24** (1), 9–16. 10.1080/1023697X.2016.1262292 (2017).

[16] Xu, H. et al. Experimental study on the wave attenuation performance of a floating breakwater under oblique irregular waves and wave–current interaction conditions. *Front. Mar. Sci.* **13**10.3389/fmars.2026.1866127 (2026).

[17] Ji, X., Gao, X., Jia, W., Tang, A. & Zou, L. Experimental study on wave attenuation performance and motion responses of a water ballast type floating breakwater. *Ocean Eng.* **359**, 126030. 10.1016/j.oceaneng.2026.126030 (2026).

[18] Dong, G. H. et al. Experiments on wave transmission coefficients of floating breakwaters. *Ocean Eng.* **35** (8–9), 931–938 (2008).

[19] Ji, C. Y., Chen, X., Cui, J., Yuan, Z. M. & Incecik, A. Experimental study of a new type of floating breakwater. *Ocean Eng.* **105**, 295–303 (2015).

[20] Al-Sairafi, F. A., Zhang, J., Jiang, C., Almansour, A. I. & Saleh, B. Enhancing Hydrodynamic Performance of Floating Breakwaters Using Wing Plates. *Water* **16** (13), 1779 (2024).

[21] Shen, Y., Pan, J., Zhou, Y. & Wang, X. Experimental study on wave attenuation performance of a new type of floating breakwater with twin pontoons and multi porous vertical plates. *China Ocean. Eng.* **36** (3), 384–394 (2022).

[22] Paotonan, C., Umar, H., Rahman, S. & Rachman, T. Analytical Approach of Wave Transmission Coefficient through on Composite Hanging Breakwater. *IOP Conference Series: Earth and Environmental Science*, *972*(1). (2022). 10.1088/1755-1315/972/1/012077

[23] Ridlwan, A., Armono, H. D., Rahmawati, S. & Tuswan, T. Transmission Coefficient Analysis of Notched Shape Floating Breakwater Using Volume of Fluid Method: A Numerical Study. *Kapal: Jurnal Ilmu Pengetahuan Dan. Teknologi Kelautan*. **18** (1), 41–50 (2021).

[24] Panda, A., Karmakar, D. & Rao, M. Performance Evaluation of Pile-Restrained Floating Breakwater using the Multi-domain Boundary Element Method. *Journal Mar. Sci. Application*, 1–24. (2025).

[25] Wu, S. X. et al. Experimental study on a new floating breakwater with openings, arc-shaped wings, and plates. *Physics Fluids*, **36**(7). (2024).

[26] Guo, J., Zhang, Y., Bian, X. & Xu, S. Hydrodynamic performance of a multi-module three-cylinder floating breakwater system under the influence of reefs: A 3D experimental study. *J. Mar. Sci. Eng.* **9** (12), 1364 (2021).

[27] Song, F., Cheng, Y., Dai, S., Yuan, Z. & Incecik, A. Hydrodynamic characteristics and parametric optimizations of a hybrid system combining a hinged wave energy converter and a breakwater with a semi-opened moonpool. *Energy. Conv. Manag.* **313**, 118614 (2024).

[28] van der Zanden, J. et al. Wave basin tests of a multi-body floating PV system sheltered by a floating breakwater. *Energies*, *17*(9), 2059. (2024).

[29] Zhao, X. L., Ning, D. Z., Zou, Q. P., Qiao, D. S. & Cai, S. Q. Hybrid floating breakwater-WEC system: A review. *Ocean Eng.* **186**, 106126 (2019).

[30] Leng, S., Xue, S., Jin, Y., Xu, G. & Xie, W. Wave dissipation effect of a new combined breakwater and its protective performance for coastal box girder bridges. *Adv. Bridge Eng.* **5** (1), 18 (2024).

[31] Lyu, F., Ji, C., Yan, M., Wang, M. & Xu, S. Experimental study on the hydrodynamic performance of an induced wave-breaking floating breakwater. *Ocean Eng.* **363**, 126660. 10.1016/j.oceaneng.2026.126660 (2026).

[32] ElKotby, M. R., Abouelsaad, O. & Masria, A. Implications of surface modifying on seawall hydrodynamics performance: Reflection and run-up / down. *Estuar. Coast. Shelf Sci.* **329**10.1016/j.ecss.2025.109666 (2026).

[33] Koraim, A. S., Heikal, E. M. & Abo Zaid, A. A. Hydrodynamic characteristics of porous seawall protected by submerged breakwater. *Appl. Ocean Res.* **46**, 1–14. 10.1016/j.apor.2014.01.003 (2014).

[34] Goda, Y. & Suzuki, Y. Estimation of incident and reflected waves in random wave experiments. In *Coastal Engineering 1976* (pp. 828–845). (1976).

[35] Hamed, B. et al. Assessing the impact of novel hybrid floating breakwater-WEC systems on hydrodynamic performance and sustainable energy outputs. *Sci. Rep.* **16** (1), 7189. 10.1038/s41598-026-37290-8 (2026). 41702959 10.1038/s41598-026-37290-8 PMC12920634

[36] Schmitt, P. & Elsäßer, B. The application of Froude scaling to model tests of Oscillating Wave Surge Converters. *Ocean Eng.* **141**, 108–115. 10.1016/j.oceaneng.2017.06.003 (2017).

[37] Heller, V. Scale effects in physical hydraulic engineering models. *J. Hydraul. Res.* **49** (3), 293–306. 10.1080/00221686.2011.578914 (2011).

